# Metformin prevents aggressive ovarian cancer growth driven by high-energy diet: similarity with calorie restriction

**DOI:** 10.18632/oncotarget.3434

**Published:** 2015-03-26

**Authors:** Zaid Al-Wahab, Ismail Mert, Calvin Tebbe, Jasdeep Chhina, Miriana Hijaz, Robert T. Morris, Rouba Ali-Fehmi, Shailendra Giri, Adnan R. Munkarah, Ramandeep Rattan

**Affiliations:** ^1^ Wayne State University, Detroit, MI; ^2^ Division of Gynecologic Oncology, Department of Women's Health, Henry Ford Hospital, Detroit, MI; ^3^ Department of Pathology, Karmanos Cancer Institute, Wayne State University, Detroit, MI; ^4^ Department of Neurology, Henry Ford Hospital, Detroit, MI; ^5^ Josephine Cancer Institute, Henry Ford Hospital, Detroit, MI

**Keywords:** ovarian cancer, metformin, calorie restriction, AMPK, mTOR

## Abstract

Caloric restriction (CR) was recently demonstrated by us to restrict ovarian cancer growth *in vivo*. CR resulted in activation of energy regulating enzymes adenosine monophosphate activated kinase (AMPK) and sirtuin 1 (SIRT1) followed by downstream inhibition of Akt-mTOR. In the present study, we investigated the effects of metformin on ovarian cancer growth in mice fed a high energy diet (HED) and regular diet (RD) and compared them to those seen with CR in an immunocompetent isogeneic mouse model of ovarian cancer. Mice either on RD or HED diet bearing ovarian tumors were treated with 200 mg/kg metformin in drinking water. Metformin treatment in RD and HED mice resulted in a significant reduction in tumor burden in the peritoneum, liver, kidney, spleen and bowel accompanied by decreased levels of growth factors (IGF-1, insulin and leptin), inflammatory cytokines (MCP-1, IL-6) and VEGF in plasma and ascitic fluid, akin to the CR diet mice. Metformin resulted in activation of AMPK and SIRT1 and inhibition of pAkt and pmTOR, similar to CR. Thus metformin can closely mimic CR's tumor suppressing effects by inducing similar metabolic changes, providing further evidence of its potential not only as a therapeutic drug but also as a preventive agent.

## INTRODUCTION

Epithelial ovarian cancer affects more than 239 000 women, with 152 000 deaths annually worldwide [1]. In 2014, 21 980 women were estimated to be diagnosed with ovarian cancer and 14 270 died from this disease, making ovarian cancer the fifth leading cause of cancer death in women in the United States [2]. This high mortality rate is due to diagnosis at an advanced stage signifying widespread metastasis within the abdomen [3]. Cytoreductive surgery and chemotherapy with platinum and taxanes have increased the disease-free and overall survival, but recurrence of the disease is common in these patients [4].

Energy balance is defined as the balance between caloric intake and expenditure [5] and has been associated with the pathogenesis of various cancers. Caloric restriction (CR) has been demonstrated to attenuate tumorogenesis in various animal models [6]; on the other hand, a positive energy state and obesity has been demonstrated to be a contributing factor for multiple cancers including gynecological cancers, such as endometrium [7], ovary [8] and breast [9], as well as non-gynecological cancers, including pancreas [10], liver [11] and colon cancer [12]. In a previous study, we showed that a high energy diet (HED) was associated with extensive ovarian tumor formation, elevation of insulin, insulin growth factor (IGF-1) and higher levels of inflammation markers in an isogeneic mouse model of ovarian cancer. Conversely, CR diet exhibited less tumor burden with a significant reduction in levels of insulin, IGF-1 and inflammation markers [13]. The underlying biologic mechanisms of calorie intake and tumorogenic/anti-tumorogenic effects are not fully understood, but it has been shown to be associated with modulation of metabolic enzymes like adenosine monophosphate activated kinase (AMPK) and sirtuin 1 (SIRT1) [14, 15]. AMPK is a heterotrimeric serine/threonine protein kinase that acts as an ultrasensitive cellular energy sensor maintaining the energy balance within the cell [16] and has been shown to have a role in inhibiting the proliferation of tumor cells [17]. AMPK performs its anti-tumorogenic activity in multiple ways including cell cycle arrest associated with stabilization of p53 and cyclin-dependent kinase inhibitors [18–20] and inhibition of cell growth by suppressing the synthesis of cellular macromolecules, including fatty acids, triglycerides, cholesterol, glycogen, ribosomal RNA and proteins [21, 22]. Mechanistically, AMPK inhibits the pro-oncogenic mammalian target of rapamycin complex (mTOR) [16, 23] and thus hampers the translation of many proteins essential for rapid cell growth. Indirect effects of AMPK results in attenuation of the insulin/IGF-1 pathways, which are known to be upregulated in many cancers including ovarian cancer [17]. SIRT1 is a nicotinamide adenine dinucleotide (NAD^+^)-dependent histone deacetylase involved in the cell's stress adaption systems, DNA damage repair, cell metabolism and survival [24, 25]. In mammals, SIRT1 expression, has been shown to be induced by CR [15] and delay age and associated diseases, such as cancer, atherosclerosis and diabetes [26–29].

Metformin is a member of the biguanide class of antihyperglycemic agents and has been recently revealed to have anti-tumorogenic effects [30, 31]. Metformin decreases hepatic glucogenesis, increases insulin sensitivity, enhances peripheral glucose uptake, and decreases glucose absorption from the gastrointestinal tract [32]. On the cellular level, metformin inhibits mitochondrial complex 1, which interferes with oxidative phosphorylation, resulting in decreased adenosine triphosphate (ATP) production and energetic stress [33]. Epidemiologic studies have shown that metformin lowers cancer risk and improves cancer outcomes in diabetic patients when compared with patients treated with other types of antihyperglycemic agents [34, 35]. Therefore, metformin has been repurposed as therapy in gynecologic and non-gynecologic cancers [36] and is currently being evaluated in various clinical trials [36]. The most evaluated mechanism of metformin's antihyperglycemia and anti-tumor activity is the activation of AMPK [36]. Metformin has also been demonstrated to induce SIRT1 levels in hepatic cancer lines [24].

In this study, we investigated the downstream effects of metformin on ovarian cancer growth using immunocompetent mouse model under nutritional excess as well as regular balanced dietary conditions and its similarity with tumor inhibitory effects of CR to observe if metformin can be utilized in lieu of CR to limit ovarian cancer growth.

## RESULTS

### Metformin decreases the tumor burden and ascites volume

**Figure 1:**
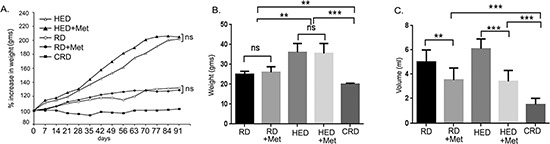
Metformin (Met) decreases the tumor burden and ascites volume Mouse ovarian tumors were generated by injecting ID8 cells in mice fed a RD, a HED and a CRD. A subset of RD and HED fed mice were treated with Met for the study period. **(A)** Average weight progression of mice per group is presented as percentage increase in weight with the average starting weight taken as 100%. **(B)** Weight at the time of sacrifice (70 days post-tumor injection). **(C)** Ascites volume as measured after collection at time of sacrifice. ***p < 0.001, **p < 0.01, ns = non-significant. CRD, caloric restriction diet; HED, high energy diet; RD, regular diet.

**Figure 2:**
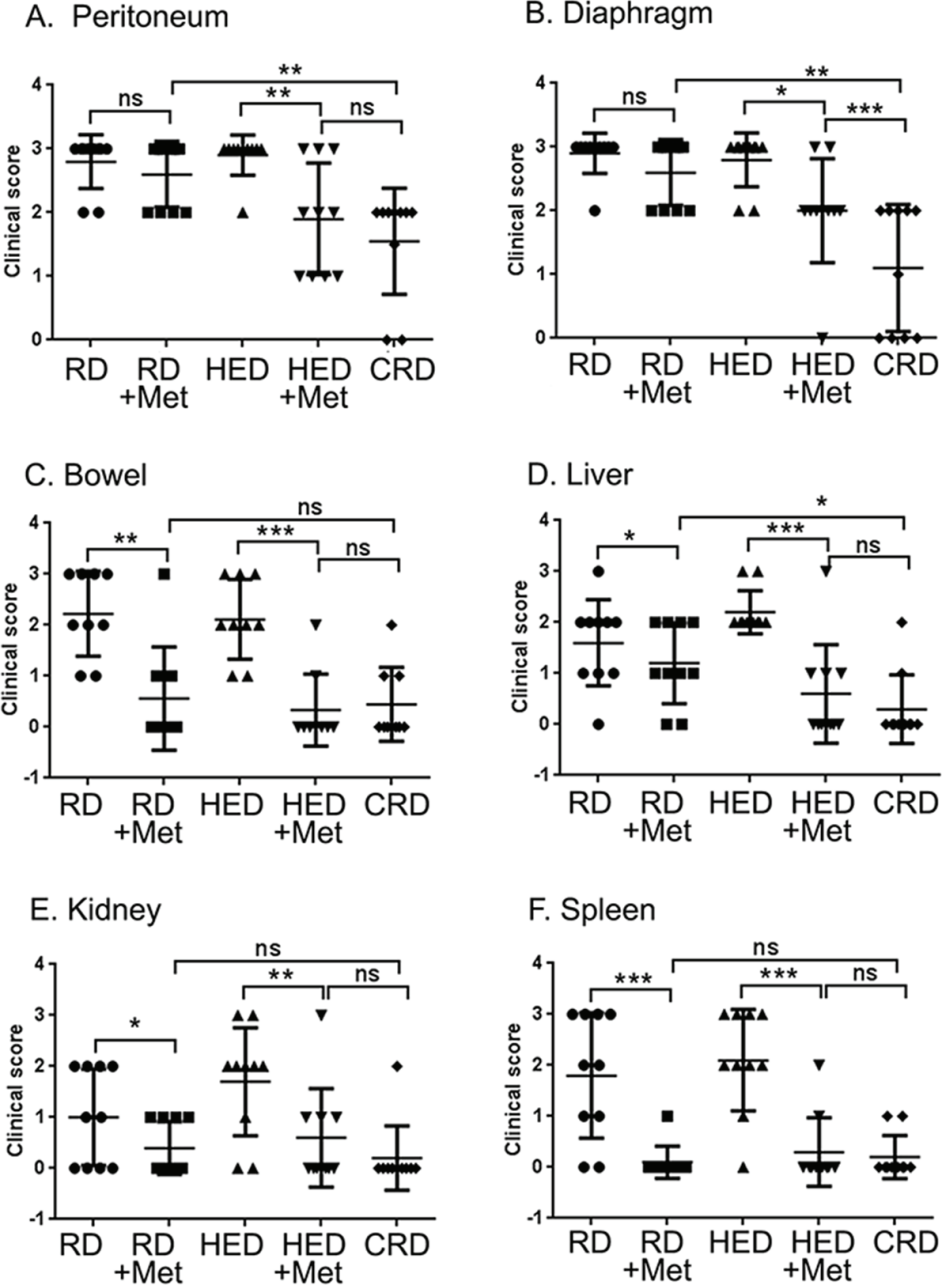
Metformin (Met) reduces the clinical ovarian tumor score At the end of the study, various organs of each mouse from the RD, HED and CRD untreated and Met treated groups were grossly examined for enumeration of visible tumor nodules. Score was stipulated as 0: no nodule; 1: 1 nodule; 2: 2 to 5 nodules and 3: more than 5 nodules observed per organ. Tumor scoring at **(A)** Peritoneum, **(B)** Diaphragm, **(C)** Bowel, **(D)** Liver, **(E)** Kidney, and **(F)** Spleen are shown. ***p < 0.001, **p < 0.01, *p < 0.05, ns = non-significant. CRD, caloric restriction diet; HED, high energy diet; RD, regular diet.

**Figure 3:**
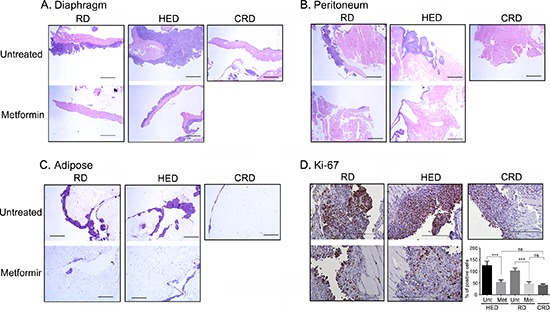
Metformin (Met) decreases the ovarian tumor growth Paraffin tumor sections obtained from the peritoneum **(A)**, diaphragm **(B)**, peritoneum and adipose were stained with hemotoxylin and eosin and visualized under a bright-field (20x) to observe for tumor nodules. Each stained tissue picture is a representative of at least 5 individual mouse sections from each of the RD, HED, CRD and Met treated groups. **(D)** Representative Ki-67 staining from the ID8 tumors at the peritoneum (200x). Count of positive Ki-67 cells from 5 high powered fields (x400) in 3 different xenografts from each group is presented as bar graph. ***p < 0.001, ns = non-significant. CRD, caloric restriction diet; HED, high energy diet; RD, regular diet; Unt, untreated.

To investigate if metformin can reduce tumor progression similar to CR, a set of HED and regular diet (RD) fed mice were given metformin daily in drinking water 7 days after tumor implantation. As previously reported [13], mice on HED had highest weight gain while the CR diet (CRD) mice maintained their weight (Figure 1A). Metformin intake did not significantly affect the weight gain of HED or RD mice (Figure 1A), which was also reflected in the end weights at the time of the sacrifice (Figure 1B). Metformin treatment significantly reduced the ascites accumulation in both RD and HED groups, but the highest reduction was observed in the CRD group (Figure 1C). Metformin treatment of the HED group was most effective in reducing the tumor progression with significant reduction of tumor burden in the peritoneum, diaphragm, bowel, liver, kidney and spleen (Figure 2A–2F). In the RD group, metformin reduced the tumor burden at the bowel, liver, kidney and spleen. CRD group had the lowest tumor burden at all organ sites (Figure 2A–2F). The hemotoxylin and eosin evaluation of the tumor sections also showed reduced tumor nodules at the diaphragm, peritoneum and adipose tissue (Figure 3A–3C) with metformin treatment in both HED and RD groups, akin to CRD group. This was also reflected in the decreased number of positive Ki-67 stained cells observed in tumors from CRD and metformin treated groups, quantified as Ki-67 index (Figure 3D). Overall, our data showed that metformin decreased the ascites and tumor burden in both the RD and HED groups significantly, similar to CRD. However, the tumor reduction by metformin in HED group was more pronounced than the tumor reduction observed in the RD treatment group.

### Metformin regulates the levels of hormones controlling the energy balance

**Figure 4:**
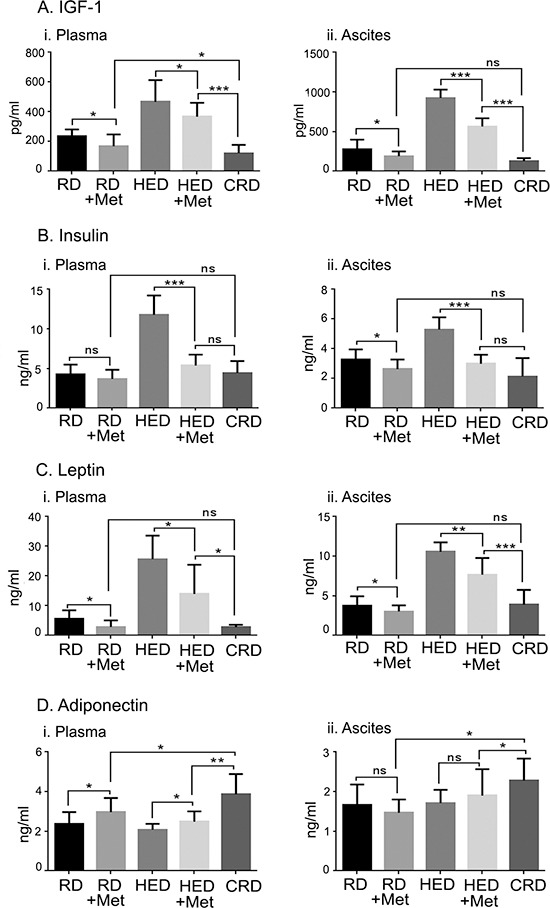
Metformin (Met) regulates the levels of hormones controlling the energy balance Plasma and ascitic fluid collected from ovarian tumor bearing mice (*n* = 6) on RD, HED, and CRD and Met on day 70 were subjected to enzyme-linked immunosorbent assay to determine the levels of **(Ai, ii)** IGF-1, **(Bi, ii)** insulin, **(Ci, ii)** leptin and **(Di, ii)** adiponectin. ***p < 0.001, **p < 0.01, *p < 0.05, ns= non-significant. CRD, caloric restriction diet; HED, high energy diet; IGF, insulin growth factor; RD, regular diet.

Growth hormones including insulin, IGF-1, leptin and adinopectin that regulate energy metabolism were estimated under metformin treatments in plasma and ascites fluid of the mice. Metformin was most efficient in reducing the levels of insulin, IGF-1 and leptin in both the plasma and ascites of the HED group (Figure 4A–4C). Adiponectin was significantly increased in the plasma but not in the ascites by metformin in the HED mice (Figure 4D). Metformin decreased IGF-1 and leptin levels significantly in both the plasma and ascites of the RD group, while insulin was reduced only in the ascites (Figure 4A–4C). Adiponectin was significantly increased in the plasma but not in the ascites by metformin in the RD mice (Figure 4D). CRD mice still had the lowest levels of IGF-1, insulin and leptin and increased adiponectin as observed previously [13]. In general, HED fed mice showed a tumor promoting environment while mice on CRD showed the inverse profile. Metformin reversed most of these tumor promoting effects of diet in HED and RD, similar to the CRD group; however, CRD was the most effective in maintaining the lowest levels of all growth factors and hormones.

### Metformin decreased the inflammatory markers and angiogenic factors

**Figure 5:**
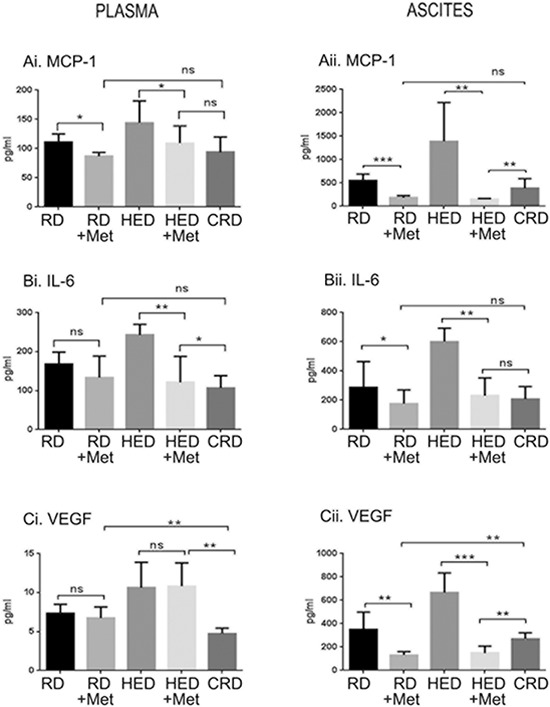
Metformin (Met) decreased the inflammatory markers and angiogenic factors Plasma and ascitic fluid collected from ovarian tumor bearing mice (*n* = 6) on RD, HED, and CRD and Met on day 70 were subjected to enzyme-linked immunosorbent assay to determine the levels of **(Ai, ii)** MCP-1, **(Bi, ii)** IL-6 and **(Ci, ii)** VEGF. ***p < 0.001, **p < 0.01, *p < 0.05, ns = non-significant. CRD, caloric restriction diet; HED, high energy diet; IL-6, interleukin 6; MCP-1, monocyte chemoattractant protein-1; RD, regular diet; VEGF, vascular endothelial growth factor.

The role of inflammatory molecules (monocyte chemoattractant protein-1 [MCP-1] and interleukin 6 [IL-6]) and angiogenic factors (vascular endothelial growth factor [VEGF]) in ovarian tumorogenesis is well established [37, 38], and the inhibition of these markers by CRD has been recently demonstrated [13]. As observed with growth factor levels, metformin had more pronounced inhibition of these factors in HED group compared to the RD group. Excluding plasma VEGF (Figure 5Ci), the levels of MCP-1, IL-6 and VEGF were significantly reduced by metformin (Figure 5A, 5B, 5Cii). Metformin did not reduce IL-6 and VEGF in the plasma of RD mice but significantly reduced MCP-1 (Figure 5Ai, Bi, 5Ci), while all 3 were significantly inhibited in the ascitic fluid (Figure 5Aii, 5Bii, 5Cii). The CRD group had lower levels of MCP-1, IL-6 and VEGF compared to the HED and RD groups. Interestingly, metformin decreased MCP-1 and VEGF levels in the ascites of the RD and HED groups more significantly than CR (Figure 5Aii, 5Cii). Based on these results, it can be suggested that metformin significantly alters the inflammatory and angiogenic armamentarium of ovarian cancer cells, even under the conditions of rich nutrition.

### Metformin induced AMPK and SIRT1

**Figure 6:**
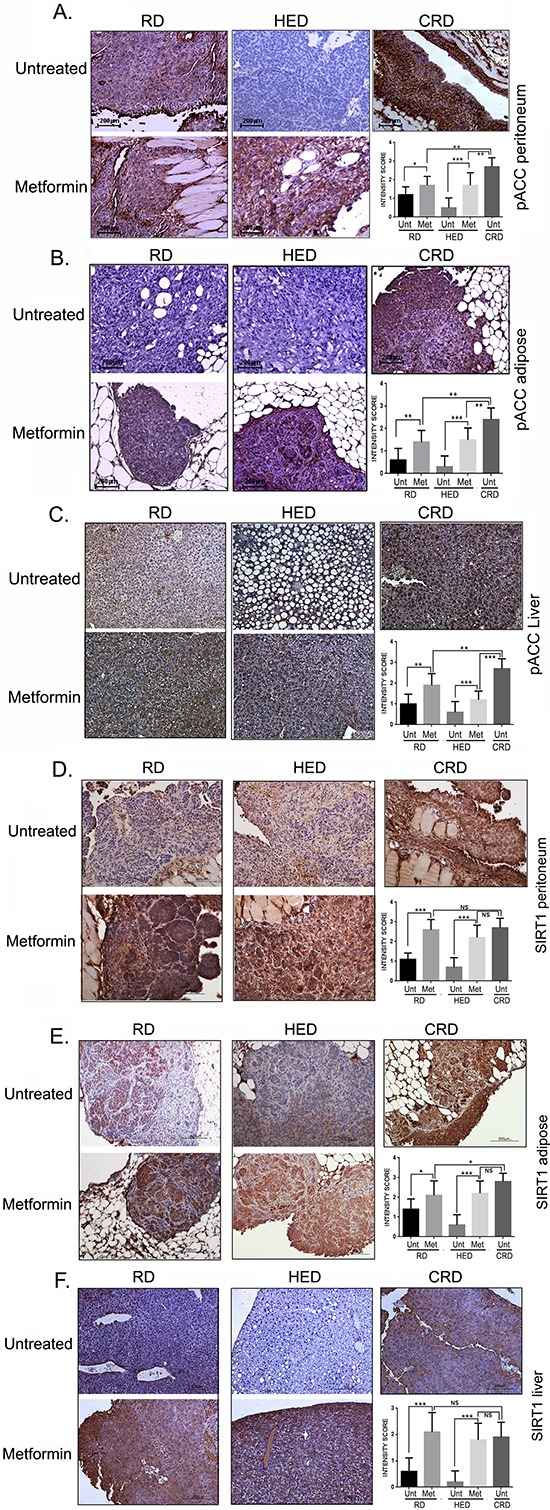
Metformin (Met) induced AMPK and SIRT1 Paraffin tumor sections obtained from the peritoneum and adipose sites and liver of mice from the RD, HED, and CRD groups with and without Met treatment were immunostained with antibodies against phosphorylated acetyl-CoA carboxylase (pACC), as a marker for AMPK activation **(A, B, C)** and SIRT1 **(D, E, F)**. Stains were developed using chromogen and visualized under a bright-field (200x) to observe for positive brown stain indicative of expression. Each stained tissue picture is a representative of at least 5 different fields examined per section from a minimum of 3 individual stained sections per group. Quantification of the intensity of the stain was performed on a scale of 0–3: 0–1 for no or weak stain; 2 for moderate stain and 3 for strong stain from 3 different fields of a minimum of 2 stained sections and is represented as a bar graph. ***p < 0.001, **p < 0.01, *p < 0.05, ns = non-significant. AMPK, adenosine monophosphate activated kinase; CRD, caloric restriction diet; HED, high energy diet; RD, regular diet; SIRT1, sirtuin 1; Unt, untreated.

AMPK and SIRT are 2 enzymes involved in regulation of energy metabolism and reported to mediate the positive effects of CR [16, 25]. The CRD group showed the strongest activation of phosphorylated acetyl-CoA carboxylase (pACC), a surrogate marker for AMPK activation, while HED groups demonstrated almost no phosphorylation of ACC as reported before [13] (Figure 6A, 6B). Metformin increased the pACC expression significantly in RD and HED groups in both peritoneal and adipose tumor tissue. A similar pattern was also observed for SIRT1 expression in the tumor tissues (Figure 6D, 6E). Metformin treatment also activated AMPK and SIRT1 in the liver, which was also associated with the amelioration of the hepatic steatosis observed in the HED group (Figure 6C, 6F). The quantification of the staining intensity is represented as bar graphs (0–1: no or weak stain; 2: moderate stain and 3: strong stain), respective of each panel. Taken together, these results suggest that metformin activated AMPK and increased the expression of SIRT1 significantly, parallel to CRD both in the tumor and in the host tissue.

### pAkt and p-mTOR expression were inhibited by metformin

**Figure 7:**
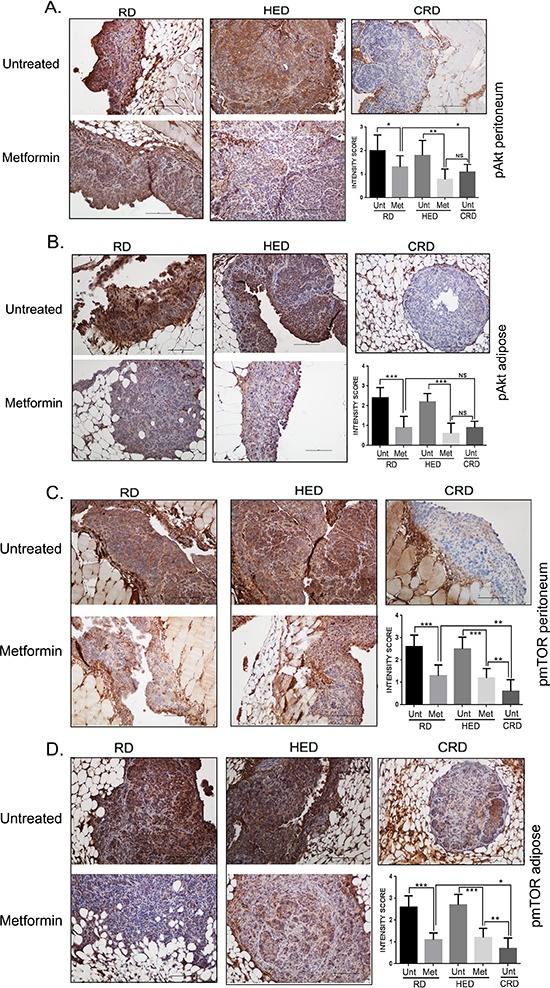
Metformin (Met) inhibited pAKT and pmTOR Paraffin tumor sections obtained from the peritoneum and adipose sites of mice from the RD, HED and CRD groups with and without Met treatment were immunostained with antibodies against phosphorylayed protein kinase B (pAkt) **(A, B)** and mammalian target of rapamycin (p-mTOR) **(C, D)**. Stains were developed using chromogen and visualized under a bright-field (200x) to observe for positive brown stain indicative of expression. Each stained section is a representative of at least 5 different fields examined per section from a minimum of 3 individual stained sections per group. Quantification of the intensity of the stain was performed on a scale of 0–3: 0–1 for no or weak stain; 2 for moderate stain and 3 for strong stain from 3 different fields of minimum of 2 stained sections and is represented as a bar graph. ***p < 0.001, **p < 0.01, *p < 0.05, ns = non-significant. AMPK, adenosine monophosphate activated kinase; CRD, caloric restriction diet; HED, high energy diet; RD, regular diet; SIRT1, sirtuin 1; Unt, untreated.

Phosphorylated protein kinase B (pAkt) and phosphorylated mTOR (p-mTOR), are the 2 common downstream signaling molecules for action of CRD, AMPK and SIRT1 [21, 36, 39–41]. CRD mice had the lowest and HED and RD mice had the highest expression of pAkt (Figure 7A, 7B) and p-mTOR (Figure 7C, 7D) as reported previously [13]. Metformin treatment reduced both pAkt and p-mTOR expression in HED and RD groups in the peritoneal and adipose tissue, similar to CRD (Figure 7A–7D). The quantification of the staining intensity is represented as bar graphs. This demonstrates that metformin modulated common oncogenic factors with CR, even under HED conditions.

## DISCUSSION

Our study shows that the levels of caloric intake have significant effects on ovarian cancer growth and these effects can be modulated by metformin. We revalidate that the positive energy balance provided by HED aggravates ovarian cancer by creating an environment that encourages cancer growth, while a negative energy balance achieved by 30% CR modulated the progression of ovarian tumors in mice. Here, we have demonstrated that metformin inhibits ovarian cancer growth by reorganizing the tumor promoting parameters under a high energy state in a similar manner as that achieved by a CRD.

Obesity has been suggested to be a prognostic factor for ovarian cancer. Having a body mass index of more than 35 is associated with an increased risk of mortality in ovarian cancer patients with a relative risk of 1.5 [8]. In another study, being overweight or obese in *early* adulthood is associated with a higher mortality among patients with ovarian cancer [42]. Metformin intake has been shown to decrease ovarian cancer risk in diabetic patients [43], increase progression free survival [44] and overall survival in women with ovarian cancer [45]. While there are likely more than one mechanism for these improved outcomes and inhibition of ovarian cancer tumorogenesis, one of the mechanisms might be the regulation of deranged host energy balance by metformin related to adiposity, deregulated insulin-IGF-1 pathway or chronic inflammation, which is often observed in diabetic and cancer patients [46]. Increased energy balance, which culminates in increased adiposity, changes the levels of hormones such as insulin, adiponectin, leptin and IGF-1 [47], which is also associated with cancer including ovarian [48, 49]. Insulin has tumor-enhancing effects and exerts these effects directly via insulin or indirectly via IGF-1 receptors on preneoplastic and neoplastic cells or other growth receptors [50], most frequently resulting in activation of the P13K/Akt-mTOR pathway, a central regulator of cell growth, proliferation and survival [6, 51, 52]. On the other hand, decreased adiponectin level has been associated with the development of colorectal [53], endometrium [54] and breast cancer [55]. Metformin modifies these hormones and growth factor levels in ovarian cancer-bearing mice fed HED or RD, which could ultimately decrease the tumor burden. An interesting observation is that metformin was the most efficient in reducing insulin and IGF-1 levels in the HED group, consistent with the highest tumor reduction by metformin observed in the HED group. This might be secondary to the fact that HED caused the most significant metabolic and hormonal derangements, and metformin might be more effective in a milieu where these derangements are more profound, as opposed to RD. Similarly, metformin also showed reduction in IL-6, MCP-1 and VEGF levels, important factors shown to promote ovarian tumor progression [56–60]. MCP-1 was reduced most significantly by metformin, which was also observed in our previous study, where metformin negated the effect of adipocyte mediated MCP-1 production [61].

PI3K/Akt-mTOR pathway, one of the most upregulated pathway observed in ovarian and other cancers, is also the common downstream pathway implicated in growth factor signaling and CR [62–64]. Akt stimulates the cell cycle progression, cell survival and inhibits apoptosis [63, 65]. mTOR is a nutrition regulated serine/threonine protein kinase that is activated by the Akt pathway but is inhibited by AMPK [66–68]. AMPK is a serine/threonine protein kinase that acts as a sensor of cellular energy status and is regulated by the AMP:ATP levels [69]. Recent evidence suggests that AMPK also regulates cell proliferation, cell growth, and autophagy [70]. Our current and previous data showed that CR diet increases AMPK activation along with decreased Akt and mTOR activation. The same was achieved in the HED and RD groups with metformin treatment. This is consistent with previous studies that have demonstrated that under nutrient-deprived conditions, which result in energy depletion, AMPK is activated and transmits energetic stress signals to mTOR via tuberous sclerosis 2 and raptor, which ultimately inhibit mTOR activity [71]. SIRT1, another energy regulating enzyme, mediates the longevity conferred by CR [15, 72]. SIRT1 also regulates IGF-1, adiponectin and insulin levels in various tissues [73]. In cancer pathogenesis; SIRT1 plays a bivalent role; functional loss of SIRT1 promotes tumorogenesis because of genomic instability [25]. However, SIRT1 has also been associated with survival and proliferation of tumors such as breast [74], gastric [75] and prostate cancer [76]. Metformin treatment increased SIRT1 expression in RD and HED groups similar to the expression increased by CRD. Molecular mechanism of regulation and interaction of both these energy sensors has yet to be elucidated, but recent data have shed a light on the interaction between the AMPK and SIRT pathway [77, 78]. NAD^+^ synthesis has been claimed to be a cellular mechanism to increase sirtuin activity [77]. For instance, activation of hepatic AMPK by metformin induces increased expression of nicotinamide phosphoribosyltransferase, which increases intracellular NAD^+^/NADH ratio and ultimately activates SIRT1 [78]. On the other hand, AMPK does not affect the nicotinamide phosphoribosyltransferase enzyme in endothelial cells [79], which suggests that AMPK and SIRT1 use different interaction pathways in various tissues. To better understand the communication mechanisms between these 2 energy sensors in different types of tissues and different types of malignancies, more research is required.

By modulating the dietary intake, we modulated the host metabolism and consequently the host environment, which had a significant effect on ovarian cancer progression. This was demonstrated by the presence of hepatic steatosis or deposition of fat within hepatocytes, which is mostly prevalent in obese populations [80]. Hyperinsulinemia also contributes to hepatic steatosis by increasing the expression of lipogenic enzymes and diminishing fatty-acid oxidation [81]. We observed that metformin ameliorates hepatic steatosis in the HED liver, along with an increased expression of pACC and SIRT1 (Figure 6C), a finding which is consistent with previous studies [78–80]. AMPK also shows anti-lipogenic activity by suppressing lipogenic enzymes (acetyl-CoA carboxylase, HMGCoA reductase, etc.) [82] and lipogenesis transcription factors (sterol regulatory element-binding proteins and carbohydrate response element binding protein), which subsequently decreases lipid accumulation [81].

CR is strongly linked to retarding both cancer and aging, which share many common cellular and molecular mechanisms [83]. The insulin-IGF-1 growth factor nexus and inflammatory cytokines involved in both pathologies converge down to activate mTOR, which is inhibited both by CRD and metformin [83, 84]. mTOR is known to orchestrate the process of ‘geroconversion’, whereby it places a cell into senescence [85]. Inhibition of mTOR by rapamycin or its analogs has been shown to prevent both cancer and aging in various models [23, 86, 87]. This mTOR driven senescence can result in a selective survival advantage to cancer cells [83]. We examined mTOR mediated senescence in our model system by staining for ɣH2A.X, a known senescence biomarker [88, 89]. Nuclear expression of ɣH2A.X represents DNA damage associated with aging [88]. We could only detect its limited expression in HED tumors, while hardly any expression was seen in tumor tissue of RD and none was detected in CRD tumor tissue (Supplementary Figure S1). This indicates that HED, which resulted in hyperinsulinemia and increased activation of p-mTOR, could also trigger senescence in cancer cells harbored by the host. Further studies are required to delineate the exact role of mTOR driven senescence under high energy or CR environments in the tumor cells. Presently, the relationship between senescence and cancer is very complex as cellular senescence also serves to block tumorigenesis [90].

**Figure 8:**
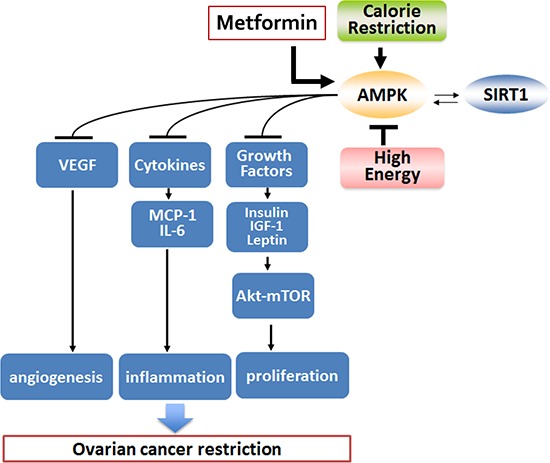
Proposed mechanism of metformin effect on ovarian cancer Metformin and CR act in a similar manner to curtail ovarian cancer growth, while HED aggravates ovarian cancer. Both metformin and CR result in activation of AMPK and SIRT1. SIRT1 activation could occur independently or as a consequence of AMPK activation. Activation of these metabolic enzymes is linked to reduction in growth factors and hormones like insulin, IGF-1 and leptin, which leads to inhibition in the protein kinase B-mammalian target of rapamycin (Akt-mTOR) pathway activation resulting in decreased ovarian tumor growth. Inhibition of cytokines like MCP-1 and IL-6 and the angiogenic factor VEGF reduces inflammation and angiogenesis, which also leads to limited ovarian tumor growth. Overall, metformin and CR have very similar effects in modulating the deranged tumor environment milieu and in effect restricts ovarian cancer progression. AMPK, adenosine monophosphate activated kinase; CR, caloric restriction; HED, high energy diet; IGF-1, insulin growth factor 1; IL-6, interleukin 6; MCP-1, monocyte chemoattractant protein-1; SIRT1, sirtuin 1; VEGF, vascular endothelial growth factor.

In summary, there are multiple pathways involved in ovarian cancer pathogenesis which metformin successfully modifies, resulting in decreased tumor formation and spread in a manner very similar to CR (Figure 8). Interestingly, metformin had a more significant effect in the high energy background suggesting an ability of metformin to be more effective in an increased metabolically deranged tumor environment. Our study also suggests that metformin has an effect not only on the tumor environment but also on modulating the host environment by making it less conducive for tumor growth, quite similar to CR. Metformin may have utilization as a CR mimetic to ameliorate aggressive tumor growth, as implementation of CR in already debilitated cancer patients may not be feasible. Our results strongly support future clinical utilization of metformin as both a repurposing drug for the treatment of ovarian cancer and also as a potential preventative agent for select populations.

## METHODS

### Tissue culture

Dr. Keith Knutson (Vaccine & Gene Therapy Institute of Florida, Port Saint Lucie, FL) donated ID8 mouse ovarian cancer cells, and they were tested for absence of standard mouse pathogen panel (Missouri University Research Animal Diagnostic Laboratory; Columbia, MO). We maintained ID8 cells in Rosewell Park Memorial Institute media, which was purchased from HyClone-ThermoScientific (Waltham, MA) containing 10% (v/v) fetal bovine serum (BioAbChem, Ladson, SC).

### Animal studies

All animal experiments were performed according to an Institutional Animal Care and Use Committee of Henry Ford Health Systems approved protocol, and institutional guidelines for the proper and humane use of animals in research were followed. Our facility is approved by the Association for Assessment and Accreditation of Laboratory Animal Care. C57B6 mice were purchased from Jackson Laboratories (Bar Harbor, ME).

#### Mouse diet

We purchased various mouse diets from Bioserv (Frenchtown, NJ). The mice groups were fed on purified RD or nutritionally balanced HED with 60% kilocalories from fat (35.7% carbohydrate; 20.5% protein) or 30% nutritionally supplemented CRD as described before [91, 92].

#### Tumor generation

Six-week-old female C57B6 mice were weighed and randomized into the 3 dietary treatment groups as described above: (1) RD (*n* = 20), (2) HED (*n* = 20) and (3) CRD (*n* = 10). Mice were weighed twice a week. After 30 days of respective diet, 5 × 10^6^ ID-8 mouse ovarian cancer cells in 200 μl phosphate-buffered saline were injected into the peritoneal cavity of the mice [13]. Post-7 days of tumor implantation, 1 set of mice from the RD and HED groups (*n* = 10) was given 200 mg/body weight metformin in water [93] daily, till the end of the study. The mice were monitored daily for any discomfort and weighed twice a week. The diet regimens were continued for another 60 days, after which the mice were sacrificed and autopsied. Ascitic fluid, blood, tumor tissue and vital organs were collected from each mouse.

### Tumor score

Tumor nodules morphology and count were identified grossly at the liver, spleen, kidneys, bowel, peritoneum and diaphragm. A scoring system to identify the tumor burden in every organ was used as 0: no nodule observed; 1: 1 nodule observed; 2: 2 to 5 nodules and 3: more than 5 nodules observed per organ [13]. Scoring was performed in a blinded manner by 2 individuals including a gynecology oncology fellow (ZW).

### Enzyme-linked immunosorbent assay (ELISA)

Levels of leptin, adiponectin, insulin, IGF-1, IL-6, VEGF and MCP-1 were estimated by enzyme-linked immunosorbent assay (ELISA) in plasma and ascitic fluid. The insulin ELISA was purchased from Millipore (Billerica, MA) and all other kits were from R&D System (Minneapolis, MN). The ELISAs were carried out according to the manufacturers' instructions.

### Immunohistochemistry

After excising the tumor tissues and other organ tissue from mice, specimens were fixed in 10% formaldehyde for 48 hours and paraffin-embedded. Consecutive sections of 4 micron thick were cut and processed for hematoxylin and eosin staining; immunohistochemistry for pACC (cat. no. 3661, used at 1:100), p-mTOR (cat. No: 2976, used at 1:50), pAkt (Ser473, cat. No: 4060 used at 1:50), SIRT1 (cat no: 15404, used at 1:100) and Ki-67 (cat. No: ab15580, used at 1:100), ɣH2A.X (cat no:9718, used at 1:100). Antibodies to pACC, p-mTOR, pAkt, and ɣH2A.X were from Cell Signaling Technology (Denver, MA). Ki-67 was from Abcam (Cambridge, MA). p16 was from Proteintech (cat. No: 10883–1-AP, used at 1:200; Proteintech Group, Chicago, IL). SIRT1 was from Santa Cruz Biotech (Santa Cruz, CA). Solutions obtained from Dako Cytomation (Carpinteria, CA) were used for performing immunostaining as described before [13, 93]. The slides were examined under a light microscope, and representative pictures were taken from a minimum of 3 slides of each group [93]. The quantification of the stain intensity was performed by assigning a score of 0–1 for no or weak stain; 2 for moderate stain and 3 for strong stain. All slides were examined in a blinded manner by 2 individuals, including a pathologist (RA).

### Statistical analysis

Data were statistically analyzed using the Graph Pad Prism software (GraphPad Software Inc, La Jolla, CA) using a combination of *t*-test and analysis of variance methods.

## SUPPLEMENTARY FIGURE



## References

[1] Ferlay J, Soerjomataram I, Dikshit R, Eser S, Mathers C, Rebelo M, Parkin DM, Forman D, Bray F (2015). Cancer incidence and mortality worldwide: Sources, methods and major patterns in GLOBOCAN 2012. Int J Cancer.

[2] Siegel R, Ma J, Zou Z, Jemal A (2014). Cancer statistics, 2014. CA Cancer J Clin.

[3] Jayson GC, Kohn EC, Kitchener HC, Ledermann JA (2014). Ovarian cancer. Lancet.

[4] Tanner EJ, Chi DS, Eisenhauer EL, Diaz-Montes TP, Santillan A, Bristow RE (2010). Surveillance for the detection of recurrent ovarian cancer: survival impact or lead-time bias?. Gynecol Oncol.

[5] Patel AC, Nunez NP, Perkins SN, Barrett JC, Hursting SD (2004). Effects of energy balance on cancer in genetically altered mice. J Nutr.

[6] Hursting SD, Smith SM, Lashinger LM, Harvey AE, Perkins SN (2010). Calories and carcinogenesis: lessons learned from 30 years of calorie restriction research. Carcinogenesis.

[7] Austin H, Austin JM, Partridge EE, Hatch KD, Shingleton HM (1991). Endometrial cancer, obesity, and body fat distribution. Cancer Res.

[8] Calle EE, Rodriguez C, Walker-Thurmond K, Thun MJ (2003). Overweight, obesity, and mortality from cancer in a prospectively studied cohort of U.S. adults. N Engl J Med.

[9] Malin A, Matthews CE, Shu XO, Cai H, Dai Q, Jin F, Gao YT, Zheng W (2005). Energy balance and breast cancer risk. Cancer Epidemiol Biomarkers Prev.

[10] Yuan C, Bao Y, Wu C, Kraft P, Ogino S, Ng K, Qian ZR, Rubinson DA, Stampfer MJ, Giovannucci EL, Wolpin BM (2013). Prediagnostic body mass index and pancreatic cancer survival. J Clin Oncol.

[11] Chen Y, Wang X, Wang J, Yan Z, Luo J (2012). Excess body weight and the risk of primary liver cancer: an updated meta-analysis of prospective studies. Eur J Cancer.

[12] Giovannucci E, Ascherio A, Rimm EB, Colditz GA, Stampfer MJ, Willett WC (1995). Physical activity, obesity, and risk for colon cancer and adenoma in men. Ann Intern Med.

[13] Al-Wahab Z, Tebbe C, Chhina J, Dar SA, Morris RT, Ali-Fehmi R, Giri S, Munkarah AR, Rattan R (2014). Dietary energy balance modulates ovarian cancer progression and metastasis. Oncotarget.

[14] Kim TH, Suh DH, Kim MK, Song YS (2014). Metformin against cancer stem cells through the modulation of energy metabolism: special considerations on ovarian cancer. Biomed Res Int.

[15] Wang Y (2014). Molecular links between caloric restriction and sir2/SIRT1 activation. Diabetes Me.

[16] Hardie DG (2007). AMP-activated protein kinase as a drug target. Annu Rev Pharmacol Toxicol.

[17] Hardie DG, Alessi DR (2013). LKB1 and AMPK and the cancer-metabolism link - ten years after. BMC Biol.

[18] Imamura K, Ogura T, Kishimoto A, Kaminishi M, Esumi H (2001). Cell cycle regulation via p53 phosphorylation by a 5′-AMP activated protein kinase activator, 5-aminoimidazole-4- carboxamide-1-beta-D-ribofuranoside, in a human hepatocellular carcinoma cell line. Biochem Biophys Res Commun.

[19] Jones RG, Plas DR, Kubek S, Buzzai M, Mu J, Xu Y, Birnbaum MJ, Thompson CB (2005). AMP-activated protein kinase induces a p53-dependent metabolic checkpoint. Mol Cell.

[20] Liang J, Shao SH, Xu ZX, Hennessy B, Ding Z, Larrea M, Kondo S, Dumont DJ, Gutterman JU, Walker CL, Slingerland JM, Mills GB (2007). The energy sensing LKB1-AMPK pathway regulates p27(kip1) phosphorylation mediating the decision to enter autophagy or apoptosis. Nat Cell Biol.

[21] Hardie DG (2007). AMP-activated/SNF1 protein kinases: conserved guardians of cellular energy. Nat Rev Mol Cell Biol.

[22] Hardie DG, Ross FA, Hawley SA (2012). AMPK: a nutrient and energy sensor that maintains energy homeostasis. Nat Rev Mol Cell Biol.

[23] Blagosklonny MV (2013). Rapamycin extends life- and health span because it slows aging. Aging (Albany NY).

[24] Nelson LE, Valentine RJ, Cacicedo JM, Gauthier MS, Ido Y, Ruderman NB (2012). A novel inverse relationship between metformin-triggered AMPK-SIRT1 signaling and p53 protein abundance in high glucose-exposed HepG2 cells. Am J Physiol Cell Physiol.

[25] Roth M, Chen WY (2014). Sorting out functions of sirtuins in cancer. Oncogene.

[26] Herranz D, Munoz-Martin M, Canamero M, Mulero F, Martinez-Pastor B, Fernandez-Capetillo O, Serrano M (2010). Sirt1 improves healthy ageing and protects from metabolic syndrome-associated cancer. Nat Commun.

[27] Cohen HY, Miller C, Bitterman KJ, Wall NR, Hekking B, Kessler B, Howitz KT, Gorospe M, de Cabo R, Sinclair DA (2004). Calorie restriction promotes mammalian cell survival by inducing the SIRT1 deacetylase. Science.

[28] Finkel T, Deng CX, Mostoslavsky R (2009). Recent progress in the biology and physiology of sirtuins. Nature.

[29] Imai S, Guarente L (2010). Ten years of NAD-dependent SIR2 family deacetylases: implications for metabolic diseases. Trends Pharmacol Sci.

[30] Zakikhani M, Dowling R, Fantus IG, Sonenberg N, Pollak M (2006). Metformin is an AMP kinase-dependent growth inhibitor for breast cancer cells. Cancer Res.

[31] Isakovic A, Harhaji L, Stevanovic D, Markovic Z, Sumarac-Dumanovic M, Starcevic V, Micic D, Trajkovic V (2007). Dual antiglioma action of metformin: cell cycle arrest and mitochondria-dependent apoptosis. Cell Mol Life Sci.

[32] Kirpichnikov D, McFarlane SI, Sowers JR (2002). Metformin: an update. Ann Intern Med.

[33] Pollak MN (2012). Investigating metformin for cancer prevention and treatment: the end of the beginning. Cancer Discov.

[34] Decensi A, Puntoni M, Goodwin P, Cazzaniga M, Gennari A, Bonanni B, Gandini S (2010). Metformin and cancer risk in diabetic patients: a systematic review and meta-analysis. Cancer Prev Res (Phila).

[35] Evans JM, Donnelly LA, Emslie-Smith AM, Alessi DR, Morris AD (2005). Metformin and reduced risk of cancer in diabetic patients. BMJ.

[36] Febbraro T, Lengyel E, Romero IL (2014). Old drug, new trick: repurposing metformin for gynecologic cancers?. Gynecol Oncol.

[37] Maccio A, Madeddu C (2012). Inflammation and ovarian cancer. Cytokine.

[38] Saharinen P, Eklund L, Pulkki K, Bono P, Alitalo K (2011). VEGF and angiopoietin signaling in tumor angiogenesis and metastasis. Trends Mol Med.

[39] Carling D (2005). AMP-activated protein kinase: balancing the scales. Biochimie.

[40] Hong S, Zhao B, Lombard DB, Fingar DC, Inoki K (2014). Cross-talk between sirtuin and mammalian target of rapamycin complex 1 (mTORC1) signaling in the regulation of S6 kinase 1 (S6K1) phosphorylation. J Biol Chem.

[41] Ruderman NB, Xu XJ, Nelson L, Cacicedo JM, Saha AK, Lan F, Ido Y (2010). AMPK and SIRT1: a long-standing partnership?. Am J Physiol Endocrinol Metab.

[42] Yang HS, Yoon C, Myung SK, Park SM (2011). Effect of obesity on survival of women with epithelial ovarian cancer: a systematic review and meta-analysis of observational studies. Int J Gynecol Cancer.

[43] Bodmer M, Becker C, Meier C, Jick SS, Meier CR (2011). Use of metformin and the risk of ovarian cancer: a case-control analysis. Gynecol Oncol.

[44] Romero IL, McCormick A, McEwen KA, Park S, Karrison T, Yamada SD, Pannain S, Lengyel E (2012). Relationship of type II diabetes and metformin use to ovarian cancer progression, survival, and chemosensitivity. Obstet Gynecol.

[45] Kumar S, Meuter A, Thapa P, Langstraat C, Giri S, Chien J, Rattan R, Cliby W, Shridhar V (2013). Metformin intake is associated with better survival in ovarian cancer: a case-control study. Cancer.

[46] Kasznicki J, Sliwinska A, Drzewoski J (2014). Metformin in cancer prevention and therapy. Ann Transl Med.

[47] Perez-Hernandez AI, Catalan V, Gomez-Ambrosi J, Rodriguez A, Fruhbeck G (2014). Mechanisms linking excess adiposity and carcinogenesis promotion. Front Endocrinol (Lausanne).

[48] Calle EE, Kaaks R (2004). Overweight, obesity and cancer: epidemiological evidence and proposed mechanisms. Nat Rev Cancer.

[49] Pischon T, Nothlings U, Boeing H (2008). Obesity and cancer. Proc Nutr Soc.

[50] Hursting SD, Berger NA (2010). Energy balance, host-related factors, and cancer progression. J Clin Oncol.

[51] Renehan AG, Frystyk J, Flyvbjerg A (2006). Obesity and cancer risk: the role of the insulin-IGF axis. Trends Endocrinol Metab.

[52] Beauchamp MC, Yasmeen A, Knafo A, Gotlieb WH (2010). Targeting insulin and insulin-like growth factor pathways in epithelial ovarian cancer. J Oncol.

[53] Guffey CR, Fan D, Singh UP, Murphy EA (2013). Linking obesity to colorectal cancer: recent insights into plausible biological mechanisms. Curr Opin Clin Nutr Metab Care.

[54] Moon HS, Chamberland JP, Aronis K, Tseleni-Balafouta S, Mantzoros CS (2011). Direct role of adiponectin and adiponectin receptors in endometrial cancer: *in vitro* and *ex vivo* studies in humans. Mol Cancer Ther.

[55] Liu LY, Wang M, Ma ZB, Yu LX, Zhang Q, Gao DZ, Wang F, Yu ZG (2013). The role of adiponectin in breast cancer: a meta-analysis. PLoS One.

[56] Scambia G, Testa U, Benedetti Panici P, Foti E, Martucci R, Gadducci A, Perillo A, Facchini V, Peschle C, Mancuso S (1995). Prognostic significance of interleukin 6 serum levels in patients with ovarian cancer. Br J Cancer.

[57] Scambia G, Testa U, Panici PB, Martucci R, Foti E, Petrini M, Amoroso M, Masciullo V, Peschle C, Mancuso S (1994). Interleukin-6 serum levels in patients with gynecological tumors. Int J Cancer.

[58] Maccio A, Madeddu C (2013). The role of interleukin-6 in the evolution of ovarian cancer: clinical and prognostic implications—a review. J Mol Med (Berl).

[59] Wang Y, Li L, Guo X, Jin X, Sun W, Zhang X, Xu RC (2012). Interleukin-6 signaling regulates anchorage-independent growth, proliferation, adhesion and invasion in human ovarian cancer cells. Cytokine.

[60] Nilsson MB, Langley RR, Fidler IJ (2005). Interleukin-6, secreted by human ovarian carcinoma cells, is a potent proangiogenic cytokine. Cancer Res.

[61] Tebbe C, Chhina J, Dar SA, Sarigiannis K, Giri S, Munkarah AR, Rattan R (2014). Metformin limits the adipocyte tumor-promoting effect on ovarian cancer. Oncotarget.

[62] Engelman JA (2009). Targeting PI3K signalling in cancer: opportunities, challenges and limitations. Nat Rev Cancer.

[63] Franke TF (2008). PI3K/Akt: getting it right matters. Oncogene.

[64] Dobbin ZC, Landen CN (2013). The Importance of the PI3K/AKT/MTOR Pathway in the Progression of Ovarian Cancer. Int J Mol Sci.

[65] Brazil DP, Yang ZZ, Hemmings BA (2004). Advances in protein kinase B signalling: AKTion on multiple fronts. Trends Biochem Sci.

[66] Guertin DA, Sabatini DM (2005). An expanding role for mTOR in cancer. Trends Mol Med.

[67] Shaw RJ, Bardeesy N, Manning BD, Lopez L, Kosmatka M, DePinho RA, Cantley LC (2004). The LKB1 tumor suppressor negatively regulates mTOR signaling. Cancer Cell.

[68] Shaw RJ, Kosmatka M, Bardeesy N, Hurley RL, Witters LA, DePinho RA, Cantley LC (2004). The tumor suppressor LKB1 kinase directly activates AMP-activated kinase and regulates apoptosis in response to energy stress. Proc Natl Acad Sci U S A.

[69] Rehman G, Shehzad A, Khan AL, Hamayun M (2014). Role of AMP-activated protein kinase in cancer therapy. Arch Pharm (Weinheim).

[70] Hoyer-Hansen M, Jaattela M (2007). AMP-activated protein kinase: a universal regulator of autophagy?. Autophagy.

[71] Kim SG, Buel GR, Blenis J (2013). Nutrient regulation of the mTOR complex 1 signaling pathway. Mol Cells.

[72] Tucci P (2012). Caloric restriction: is mammalian life extension linked to p53?. Aging (Albany NY).

[73] Yang T, Fu M, Pestell R, Sauve AA (2006). SIRT1 and endocrine signaling. Trends Endocrinol Metab.

[74] Eades G, Yao Y, Yang M, Zhang Y, Chumsri S, Zhou Q (2011). miR-200a regulates SIRT1 expression and epithelial to mesenchymal transition (EMT)-like transformation in mammary epithelial cells. J Biol Chem.

[75] Cha EJ, Noh SJ, Kwon KS, Kim CY, Park BH, Park HS, Lee H, Chung MJ, Kang MJ, Lee DG, Moon WS, Jang KY (2009). Expression of DBC1 and SIRT1 is associated with poor prognosis of gastric carcinoma. Clin Cancer Res.

[76] Huffman DM, Grizzle WE, Bamman MM, Kim JS, Eltoum IA, Elgavish A, Nagy TR (2007). SIRT1 is significantly elevated in mouse and human prostate cancer. Cancer Res.

[77] Feldman JL, Dittenhafer-Reed KE, Denu JM (2012). Sirtuin catalysis and regulation. J Biol Chem.

[78] Caton PW, Nayuni NK, Kieswich J, Khan NQ, Yaqoob MM, Corder R (2010). Metformin suppresses hepatic gluconeogenesis through induction of SIRT1 and GCN5. J Endocrinol.

[79] Zu Y, Liu L, Lee MY, Xu C, Liang Y, Man RY, Vanhoutte PM, Wang Y (2010). SIRT1 promotes proliferation and prevents senescence through targeting LKB1 in primary porcine aortic endothelial cells. Circ Res.

[80] Farrell GC, Larter CZ (2006). Nonalcoholic fatty liver disease: from steatosis to cirrhosis. Hepatology.

[81] Browning JD, Horton JD (2004). Molecular mediators of hepatic steatosis and liver injury. J Clin Invest.

[82] Li Y, Xu S, Mihaylova MM, Zheng B, Hou X, Jiang B, Park O, Luo Z, Lefai E, Shyy JY, Gao B, Wierzbicki M, Verbeuren TJ, Shaw RJ, Cohen RA, Zang M (2011). AMPK phosphorylates and inhibits SREBP activity to attenuate hepatic steatosis and atherosclerosis in diet-induced insulin-resistant mice. Cell Metab.

[83] Blagosklonny MV (2011). NCI's provocative questions on cancer: some answers to ignite discussion. Oncotarget.

[84] Dowling RJ, Zakikhani M, Fantus IG, Pollak M, Sonenberg N (2007). Metformin inhibits mammalian target of rapamycin-dependent translation initiation in breast cancer cells. Cancer Res.

[85] Blagosklonny MV (2014). Geroconversion: irreversible step to cellular senescence. Cell cycle.

[86] Bezrukov LA, Dolzhenko OG, Kalancha RI, Korneva VV, Selizar VP (1988). [Effect of paternal drunkenness and smoking on child development]. Pediatriia.

[87] Comas M, Toshkov I, Kuropatwinski KK, Chernova OB, Polinsky A, Blagosklonny MV, Gudkov AV, Antoch MP (2012). New nanoformulation of rapamycin Rapatar extends lifespan in homozygous p53−/− mice by delaying carcinogenesis. Aging (Albany NY).

[88] Saretzki G (2010). Cellular senescence in the development and treatment of cancer. Curr Pharm Des.

[89] Collado M, Serrano M (2010). Senescence in tumours: evidence from mice and humans. Nat Rev Cancer.

[90] Finkel T, Serrano M, Blasco MA (2007). The common biology of cancer and ageing. Nature.

[91] Moore T, Beltran L, Carbajal S, Hursting SD, DiGiovanni J (2012). Energy balance modulates mouse skin tumor promotion through altered IGF-1R and EGFR crosstalk. Cancer Prev Res (Phila).

[92] Lanza-Jacoby S, Yan G, Radice G, LePhong C, Baliff J, Hess R (2013). Calorie restriction delays the progression of lesions to pancreatic cancer in the LSL-KrasG12D; Pdx-1/Cre mouse model of pancreatic cancer. Exp Biol Med (Maywood).

[93] Rattan R, Graham RP, Maguire JL, Giri S, Shridhar V (2011). Metformin suppresses ovarian cancer growth and metastasis with enhancement of cisplatin cytotoxicity *in vivo*. Neoplasia.

