# Supplementary material for: Metformin prevents aggressive ovarian cancer growth driven by high-energy diet: similarity with calorie restriction
Source: Oncotarget. 2015 Mar 26;6(13):10908–23. doi: 10.18632/oncotarget.3434 (PMC4484428; doi:10.18632/oncotarget.3434)
Supplement: Supplementary file 1 [file oncotarget-06-10908-s001.pdf]

SUPPLEMENTARY FIGURE

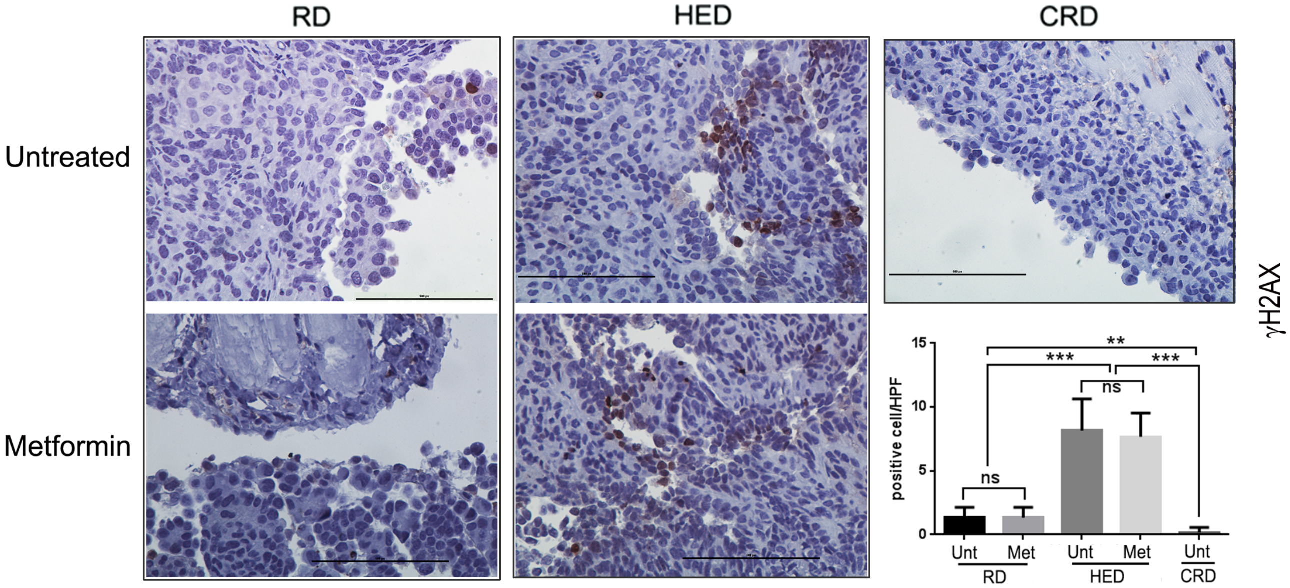

Supplementary Figure S1: Senescence maker γH2AX. Paraffin tumor sections obtained from the peritoneum sites of mice from the RD, HED and CRD groups with and without Met treatment were immunostained with antibody against γH2AX. Stains were developed using chromogen and visualized under a bright-field (400x) to observe for positive brown stain nucleus indicative of expression. Each stained section is a representative of at least 5 different fields examined per section from a minimum of 3 individual stained sections per group. Quantification of the number of positively nucleus stained cells per high power field (400x) was performed. Counts were averaged from 5 different fields of minimum of 3 stained sections per group and is represented as a bar graph. ***$p < 0.001$, **$p < 0.01$, *$p < 0.05$, ns = non-significant. CRD, caloric restriction diet; HED, high energy diet; RD, regular diet; Unt, untreated; Met, metformin.
